# Risk factors for eating disorder symptoms at 12 years of age: A 6-year longitudinal cohort study

**DOI:** 10.1016/j.appet.2016.09.005

**Published:** 2017-01-01

**Authors:** Elizabeth H. Evans, Ashley J. Adamson, Laura Basterfield, Ann Le Couteur, Jessica K. Reilly, John J. Reilly, Kathryn N. Parkinson

**Affiliations:** a Institute of Health & Society, Newcastle University, Newcastle upon Tyne, UK; b Human Nutrition Research Centre, Newcastle University, Newcastle upon Tyne, UK; c Physical Activity for Health Group, School of Psychological Sciences & Health, University of Strathclyde, Glasgow, UK

**Keywords:** Epidemiologic studies, Longitudinal studies, Eating disorders, Restraint, Body dissatisfaction, Depression, Child, Adolescent, BMI, body mass index, CBIS, Children's Body Image Scale, CDI-S, Child Depression Inventory-Short Form, ChEAT, Children's Eating Attitudes Test, DEBQ-C, Dutch Eating Behaviour Questionnaire child version, GMS, Gateshead Millennium Study, IWQOL-Kids, Impact of Weight on Quality of Life-Kids

## Abstract

Eating disorders pose risks to health and wellbeing in young adolescents, but prospective studies of risk factors are scarce and this has impeded prevention efforts. This longitudinal study aimed to examine risk factors for eating disorder symptoms in a population-based birth cohort of young adolescents at 12 years.

Participants from the Gateshead Millennium Study birth cohort (n = 516; 262 girls and 254 boys) completed self-report questionnaire measures of eating disorder symptoms and putative risk factors at age 7 years, 9 years and 12 years, including dietary restraint, depressive symptoms and body dissatisfaction. Body mass index (BMI) was also measured at each age.

Within-time correlates of eating disorder symptoms at 12 years of age were greater body dissatisfaction for both sexes and, for girls only, higher depressive symptoms. For both sexes, higher eating disorder symptoms at 9 years old significantly predicted higher eating disorder symptoms at 12 years old. Dietary restraint at 7 years old predicted boys' eating disorder symptoms at age 12, but not girls'. Factors that did not predict eating disorder symptoms at 12 years of age were BMI (any age), girls’ dietary restraint at 7 years and body dissatisfaction at 7 and 9 years of age for both sexes.

In this population-based study, different patterns of predictors and correlates of eating disorder symptoms were found for girls and boys. Body dissatisfaction, a purported risk factor for eating disorder symptoms in young adolescents, developed concurrently with eating disorder symptoms rather than preceding them. However, restraint at age 7 and eating disorder symptoms at age 9 years did predict 12-year eating disorder symptoms. Overall, our findings suggest that efforts to prevent disordered eating might beneficially focus on preadolescent populations.

## Highlights

• Body esteem and eating disorder (ED) symptoms were correlated at 12 years.
• Depressive and ED symptoms were correlated at 12 years in girls.
• Higher dietary restraint at 7 years predicted ED symptoms at 12 years in boys.
• Higher ED symptoms at 9 years predicted ED symptoms at 12 years.
• Preventive efforts for ED symptoms should begin in preadolescence.

## 1 Introduction

The incidence of eating disorders rises from childhood to early adolescence, defined as 10–13 years of age (Nicholls et al., 2011, Sawyer et al., 2012). Consequently, most previous research into the developmental psychopathology of eating disorders has begun at around 12 years old. By this age, eating disorder symptoms are already present in non-clinical populations at levels similar to those found in late adolescence (Wichstrøm, 2000), which suggests that the antecedent conditions for such disorders arise before adolescence. Eating disorder symptoms do not correspond, in severity or specificity, to full-syndrome eating disorders. Instead they encompass a broad array of dimensional maladaptive cognitions and behaviours relating to eating and weight. These cognitions and behaviours are found across the range of full-syndrome eating disorder diagnoses as well as in sub-syndromal variants (Walsh & Sysko, 2009). An understanding of causal risk factors for eating disorder symptoms is important because such symptoms increase children's risk of subsequent weight gain, depression, weight cycling and full-syndrome eating disorders in adolescence (Chamay-Weber et al., 2005, Combs et al., 2012, Field et al., 2003, Stice et al., 2000).

A recent comprehensive evidence synthesis highlighted a clear research need for prospective examinations of risk factors for disordered eating, including non-clinical samples of both boys and girls and commencing at a younger age than previous studies i.e. 6–10 years of age (Culbert, Racine, & Klump, 2015). In the current study, we prospectively examined potential risk factors, starting at age 7, to determine which variables contributed to the development of eating disorder symptoms at 12 years of age. These variables included body dissatisfaction, depression, dietary restraint, body mass index (BMI), and previous eating disorder symptoms. Given the absence of an established theoretical framework within which to situate the longitudinal development of eating disorder symptoms over preadolescence - mainly due to an absence of prospective data - these predictor variables were selected from intrapersonal risk factors for disordered eating in older adolescents and adults, broadly in line with the dual pathway model (Stice & Agras, 1998).

### 1.1 Putative predictors of eating disorder symptoms at 12 years of age

It is widely accepted that greater evaluative body dissatisfaction, also referred to as lower body esteem, contributes to the emergence and maintenance of eating disorder symptoms (Stice and Shaw, 2002, Stice, 2002). This consensus is based primarily upon findings with older, predominantly female samples. In contrast, findings with younger, mixed-sex groups are equivocal: some studies suggest that body dissatisfaction is directly correlated with, but does not predict, eating disorder symptoms over pre-adolescence, (Allen et al., 2008, Parkinson et al., 2012), instead emerging as a predictor at around 13 years old (Ferreiro, Seoane, & Senra, 2012). Conversely, other studies have found an effect in preadolescence, but only for boys (Keel, Fulkerson, & Leon, 1997) or only for girls (Gardner, Stark, Friedman, & Jackson, 2000). The effects and developmental dynamics of body dissatisfaction's relation to eating disorder symptoms may vary depending on age and gender (Culbert et al., 2015). In these terms, body dissatisfaction and eating disorder symptoms may co-emerge in pre-adolescence but the former may drive the latter during adolescence itself.

Body dissatisfaction is extremely common in preadolescence, affecting around 40% of children aged 6 to 11 (Truby & Paxton, 2008), whereas elevated eating disorder symptoms are less prevalent, (Erickson and Gerstle, 2007, Westerberg-Jacobson et al., 2012). There is a degree of conceptual and statistical overlap between the two. However, the aforementioned prevalence data suggest that it is unlikely that the former should be considered an exclusive subcategory of the latter as opposed to a potential risk factor in its own right. Further investigation is needed regarding body dissatisfaction's role as a risk factor for eating disorder symptoms in younger mixed-sex groups.

In addition to a direct relationship, body dissatisfaction may lead to eating disorder symptoms via the mediating influences of elevated depressive symptoms and greater dietary restraint (Stice, Ng, & Shaw, 2010), as proposed in the dual pathway model of eating pathology (Stice & Agras, 1998). Limited evidence suggests that depressive symptoms predict eating disorder symptoms in early adolescence among girls (Rodgers, Paxton, & McLean, 2014), although a reciprocal relationship has also been reported (Ferreiro, Wichstrøm, Seoane, & Senra, 2014). Dietary restraint has been found to predict disordered eating in adolescents (Neumark-Sztainer et al., 2006) and similarly, dietary restraint at 7 years of age was found to predict subsequent eating disorder symptoms at 9 years of age in a previous study with this cohort (Parkinson et al., 2012). It is possible that dietary restraint constitutes a precursor or subcomponent of eating disorder symptoms although, like body dissatisfaction, dietary restraint is highly prevalent (Shunk & Birch, 2004) whereas eating disorder symptoms are less so. A cross-sectional study recently demonstrated that depressive symptoms and dietary restraint fully mediated the relationship between body dissatisfaction and eating disorder symptoms in girls aged 7–11 years (Evans, Tovee, Boothroyd, & Drewett, 2013).

Higher body mass index (BMI) is another proposed risk factor for higher eating disorder symptoms, although previous studies have not found evidence of this relationship in childhood (e.g., Gardner et al., 2000, Parkinson et al., 2012) with rare exceptions (Jendrzyca & Warschburger, 2016). Research with adults and adolescents suggests that body dissatisfaction may fully mediate the inconsistently observed relationships between eating disorder symptoms and BMI (Lynch et al., 2008, Micali et al., 2015, Rohde et al., 2015, Stice and Whitenton, 2002).

Continuity of eating disorder symptoms over time has been observed amongst children and young adolescents in most previous longitudinal studies (e.g., Ferreiro et al., 2014, Gardner et al., 2000, Keel et al., 1997), suggesting that eating disorder symptoms become at least partially established at an early age (Wichstrøm, 2000). This highlights the importance of controlling for initial levels of the outcome variable (Stice, 2002) when predicting an outcome across time, something that has not always been done in previous studies of eating disorder symptoms with preadolescents (e.g., Davison, Markey, & Birch, 2003).

### 1.2 Sex differences in eating disorder symptoms

Both eating disorder symptoms and depressive symptoms are overrepresented in girls from 12 to 13 years of age onwards (Ferreiro, Seoane, & Senra, 2011), and it has been proposed that these internalising symptoms constitute female-specific reciprocal risk factors whose mutual influence escalates with time (Beato-Fernández, Rodríguez-Cano, Pelayo-Delgado, & Calaf, 2007). However, other studies suggest that depressive symptoms also play a direct causal role in boys’ eating disorder symptoms from around the age of 13 (Ferreiro et al., 2011). The influence of body dissatisfaction on eating disorder symptoms, too, may vary with sex: Ferreiro et al. (2012) found that body dissatisfaction and eating disorder symptoms were directly correlated in boys and girls at age 11 but that body dissatisfaction emerged as a causal risk factor for girls only, from age 13 onwards. However, none of these studies looked at preadolescent risk factors, and dietary restraint does not appear to have been examined as a predictor in this context. Questions remain regarding sex differences in the emergence of eating disorder symptoms, and further longitudinal studies of these phenomena in preadolescent girls and boys are clearly merited (Culbert et al., 2015).

### 1.3 Aims and hypotheses

The present study set out to examine risk factors by identifying earlier predictors and within-time correlates of eating disorder symptoms in a population-based birth cohort (Gateshead Millennium Study: GMS) of boys and girls at 12 years of age. The first aim was to identify *within-time* associations of eating disorder symptoms with measures of body dissatisfaction, depressive symptoms, and BMI all at 12 years of age. Associations were examined separately for boys and girls. The second aim was to identify *prospective predictors* of eating disorder symptoms at 12 years of age, again separately for boys and girls, taking into account prior eating disorder symptoms at 9 years of age. Putative across-time predictors included prior body dissatisfaction measured at 7 and 9 years of age, and dietary restraint measured at 7 years. BMI was also measured at 7 and 9 years.

Our expectations for the *within-time* associations (correlates) at 12 years of age were that there would be a direct association between eating disorder symptoms and body dissatisfaction for both boys and girls, based on existing findings to this effect (Ferreiro et al., 2012). In contrast, we expected that depressive symptoms would be directly associated with eating disorder symptoms for girls but not boys, based on the balance of evidence for their reciprocal relationship in girls but not boys of this age (e.g., Beato-Fernández et al., 2007). Previous research also led us to expect that BMI would be strongly directly associated with body dissatisfaction but not with disordered eating in participants of both sexes (Micali et al., 2015).

Our expectations for the *prospective* predictors of eating disorder symptoms at 12 years of age were that prior eating disorder symptoms at 9 years of age would be a strong direct predictor for both boys and girls, given evidence of continuity of such symptoms across preadolescence and beyond (Ferreiro et al., 2014). We further expected higher eating disorder symptoms at 12 years of age to be predicted by greater dietary restraint at age 7 for boys and girls, as has been found previously at 9 years of age (Parkinson et al., 2012). The balance of the existing evidence, which suggest that body dissatisfaction emerges as a causal factor at around the age of 13 years, led us to expect that body dissatisfaction at 7 or 9 years of age would not predict eating disorder symptoms at age 12 for boys or girls, acting only as a correlate (Allen et al., 2008, Ferreiro et al., 2012, Parkinson et al., 2012) rather than as a risk factor, contrary to the pattern seen in adolescent populations (Ferreiro et al., 2012).

## 2 Method

### 2.1 Participants

The data reported are from the Gateshead Millennium Study (GMS) cohort in which mothers of infants born between June 1999 and May 2000 were approached to permit their infant(s) to join a longitudinal study of feeding and growth (Parkinson, Wright, & Drewett, 2007). All infants born to mothers resident in Gateshead, an urban district in northeast England, in 34 pre-specified weeks were eligible and 1029 infants (82%) joined the study. Mothers were primarily from the white ethnic majority group (98%) (Parkinson et al., 2010), which represented the ethnic composition of the region at the time. The principal aim was to examine prospectively the joint influence of infant feeding behaviour and maternal characteristics on weight gain in a population birth cohort. Full details are published (Parkinson et al., 2007, Parkinson et al., 2010).

The cohort has been followed up at intervals since recruitment; at each follow-up assessment all children whose families had not previously asked to leave the study were eligible to participate. For the present study, assessments of the children were taken at three follow ups: 6–8 years referred to as 7 years in this paper (median 7.4, range 6.4–8.4); 8–10 years referred to as 9 years (median 9.3, range 8.4–10.2); and 11–13 years referred to as 12 years (median 12.5 range 11.6–13.3). The mean interval between the 7 and 9 year assessments was 1.9 years (*SD* = 0.2 years) and the mean time interval between the 9 and 12 year assessments was 3.2 years (*SD* = 0.3 years). Mothers gave written consent for their own participation and for the child to participate in the study. The children/adolescents gave written assent.

Favourable ethical opinions were granted by Gateshead and South Tyneside Local Research Ethics Committee (7 year follow up) and by Newcastle University Ethics Committee (9 year and 12 year follow ups).

### 2.2 Procedure

The data were collected by researchers trained in anthropometry and the other study procedures. At each follow up the children were visited in schools, or at home, to collect anthropometric and questionnaire data. If necessary the researchers helped the children with comprehension of the questionnaires, using the standardised study assessment protocol.

### 2.3 Measures

#### 2.3.1 Eating disorder symptoms and dietary restraint

i) The Dutch Eating Behaviour Questionnaire child version (DEBQ-C) (van Strien & Oosterveld, 2008) was adapted for 7–12 year olds from the Dutch Eating Behaviour Questionnaire (DEBQ) (van Strien and Oosterveld, 2008, van Strien et al., 1986). The cohort completed the seven-item Restraint subscale at 7 years old. It assesses the tendency to eat reduced amounts in order to lose or maintain weight. Participants respond ‘no’, ‘sometimes’ or ‘yes’ to each item, scoring 1, 2 or 3, with higher scores indicating greater dietary restraint. The subscale has established reliability (*α* = 0.8) and adequate construct validity in boys and girls aged 7–12 years (van Strien & Oosterveld, 2008). In the current cohort, *α* = 0.7 at the 7 year follow up assessment.

ii) The Children's Eating Attitudes Test (ChEAT) (Maloney, McGuire, & Daniels, 1988), a modified version of the adult Eating Attitudes Test (Garner and Garfinkel, 1979, Garner et al., 1982), was completed by the cohort at 9 and 12 years of age. The ChEAT is a 26-item measure of dimensional eating disorder symptoms including concerns about being overweight, binging and purging, and food pre-occupation. Items are scored between 1 (never) and 6 (always); the three most symptomatic responses are scored 1, 2 and 3 respectively, whilst the other three responses are scored zero. The scale has satisfactory test-retest reliability (*r* = 0.8) and internal consistency of *α* = 0.9 in boys and girls aged 7–12 years (Maloney et al., 1988, Smolak and Levine, 1994). Higher scores indicate greater symptomatology. Participants completed the ChEAT at 9 years and 12 years old. In the current cohort, *α* = 0.8 at 9 years and *α* = 0.8 at 12 years of age.

#### 2.3.2 Body image

i) The Children's Body Image Scale (CBIS) (Truby and Paxton, 2002, Truby and Paxton, 2008) consists of photographic figures of pre-pubescent children, seven each of boys and girls ranging from very thin to obese. The CBIS scale asks: ‘Looking at the pictures below, which body shape looks most like your own?’ (perceived figure); and ‘Looking at the same pictures, which body shape would you most like to have?’ (preferred figure). The CBIS categories were assigned scores of 1–7 to give an ordered numerical scale of increasing size. Body dissatisfaction was calculated by subtracting the perceived figure from the preferred figure to produce a directional discrepancy score, where a negative score indicated a preference for a smaller body than one's own and a positive score indicated a preference for a larger figure (Truby & Paxton, 2002). The scale demonstrates acceptable construct validity and test-retest reliability in boys and girls aged 7–11 years (*r* = 0.7). The cohort completed the CBIS at 7 years and 9 years of age.

ii) The Impact of Weight on Quality of Life-Kids (IWQOL–Kids) assesses self-perceptions of weight-specific quality of life across the entire weight spectrum for children and adolescents aged 11–19 years (Kolotkin et al., 2006). The scale is comprised of 4 sub-scales, including a 9-item body esteem subscale that assesses evaluative satisfaction with one's physical body. Respondents report the frequency with which they experience a given negative body-related cognition on a scale from 1 (always) to 5 (never). The subscale provides a reliable (*α* = 0.9) and valid measure of body esteem in overweight and non-overweight boys and girls aged 11–19 years and excellent test-retest reliability of *r* = 0.9 (Kolotkin et al., 2006, Modi and Zeller, 2011). The cohort completed the IWQOL-Kids at 12 years of age, and an internal reliability (Cronbach's *α*) of 0.9 was obtained.

The CBIS is unsuitable for 12-year-olds because the figures depict pre-pubertal children, so the age-appropriate IWQOL-Kids body esteem sub-scale was selected for the 12 year follow-up assessment. Although these scales differ in format, both capture global attitudinal body dissatisfaction (as opposed to other dimensions of body image). They are referred to as body dissatisfaction (CBIS) and body esteem (IWQOL-Kids) in the analyses to distinguish between the different measures used. Lower/more negative scores on both the CBIS and the IWQOL-Kids scale indicate greater body dissatisfaction (and thus lower body esteem).

#### 2.3.3 Depressive symptoms

The Child Depression Inventory – Short Form (CDI-S) (Kovacs, 1992) is a simplified version of the Beck Depression Inventory (Beck, Ward, Mendelson, Mock, & Erbaugh, 1961). It was used to measure depressive symptoms at 12 years. It consists of 10 items, each comprising three statements about the respondent's feelings in the preceding two weeks from which one is selected per item (scored 0, 1, or 2). A higher total score, calculated by summing each item's score, indicates greater symptomatology. The CDI-S has a satisfactory internal consistency of 0.8 in its validation sample of boys and girls aged 7–17 years (Kovacs, 1992). The internal consistency of the scale in the current sample was *α* = 0.8 at 12 years of age.

#### 2.3.4 Body measurements

Weight and height were measured by the study researchers at 7 years, 9 years and 12 years of age, using equipment purchased from Chasmors, London. Weight was measured to 0.1 kg using Tanita scales TBF-300MA, and height was measured to 0.1 cm with the head in the Frankfurt plane using a Leicester portable height measure. On each assessment occasion, measurements were taken at least twice, until two consistent values were obtained (data points within 0.1 kg of each other for weight and within 1 cm of each other for height). The mean value of the two measurements for weight and for height was calculated. The child's body mass index (BMI) (weight[kg]/height[m]^2^) was calculated from the averaged height and weight measurements. BMI *z*-scores for age were calculated using data from the UK90 reference dataset (Cole, Freeman, & Preece, 1995).

#### 2.3.5 Socio-economic status

Socio-economic status measures were collected from the mother at recruitment shortly after birth. The family's postcode was transformed into the Townsend deprivation score (Townsend, Phillimore, & Beattie, 1988). We used the Townsend deprivation score as an index of SES when evaluating the effect of attrition at 12 years (from the original cohort) upon the representativeness of the cohort.

### 2.4 Statistical methods

Data analysis was conducted on all cases for whom an eating disorder symptom score at 12 years was obtained. In total, 525 participants were assessed at the 12 year follow up, and eating disorder symptom scores were available for 516 of these (<2% missing data).

To examine the representativeness of the cohort in the light of attrition, the current cohort composition was compared to the original cohort composition according to Townsend deprivation index quintile (Table 1).

Next, descriptive statistics were calculated for all measured variables, the proportion of missing data for each variable was recorded and initial comparisons of boys' and girls’ characteristics were made (Table 2). Anthropometric and scale data were not normally distributed, so the median and the semi-interquartile range was used to summarise them, and non-parametric methods (Mann-Whitney *U* test) were used to compare values for boys and girls.

The first aim of the study was to examine *within-time* associations between eating disorder symptoms (12y) and putative correlates; correlations (Spearman's *rho*) were calculated separately for boys and girls (Table 3). A significance threshold of *p* ≤ 0.005 was applied to the data in Table 2, Table 3 to correct for multiple comparisons. Significant correlates of eating disorder symptoms (12y) were used in the subsequent multivariate regressions for boys and girls.

The second aim of the study was to develop *multivariate predictive models* for eating disorder symptoms with variables measured both within and across-time, using OLS linear regression. Disordered eating symptoms (12y) were regressed, separately for boys and girls, on variables significant in the preceding correlation analyses (Table 4). SPSS version 21 (SPSS Inc, Chicago, IL, USA) was used for statistical analysis.

## 3 Results

### 3.1 Sample attrition

The original sample comprised a total of 1011 mothers and it was comparable with the northeast region of England in terms of socio-economic deprivation apart from slight under-representation of the most affluent quintile (Table 1). Overall, non-participation has been higher in the least affluent families than in the most affluent. This means that by 12 years the distribution across all the deprivation quintiles was fairly even and the sample is representative of the north of England.

### 3.2 Descriptive statistics

Eating disorder symptom data were collected from 516 adolescents (254 boys and 262 girls) at the 12 year follow-up; 4.8% were 11 years old, 89.8% were 12 years old and 5.4% were 13 years old. Descriptive data are shown by sex at each follow up assessment (Table 2). The proportion of complete data for each variable ranged from 79 to 83% at age 7 years to 99–100% at 12 years.

At 12 years girls had significantly higher depressive symptom scores than boys and they had significantly higher body dissatisfaction than boys at 7, 9 and 12 years of age. There was no significant difference between boys and girls on the eating disorder symptom scores at either 9 or 12 years; however the scores overall at 9 years were significantly higher than at 12 years (*Z* = 10.62, *p* < 0.001). Overall, for both girls and boys, children's BMI *z*-scores at 9 years were higher than those at 7 years (*χ*^2^ = 28.4, *p* < 0.0001), and BMI *z*-scores at 12 years were higher than those at 9 years (*χ*^2^ = 13.3, *p* < 0.0001) i.e. their relative adiposity increased at each time-point.

### 3.3 Within-time associations among 12 year eating disorder symptoms and putative correlates

Table 3 shows a zero-order non-parametric correlation matrix for study variables at 12, 9 and 7 years of age. For boys, eating disorder symptoms at age 12 were inversely associated with body esteem at age 12 and directly associated with eating disorder symptoms at age 9 and dietary restraint at age 7. For girls, eating disorder symptoms at age 12 were directly associated with depressive symptoms and BMI at age 12, inversely associated with body esteem at age 12 and directly associated with eating disorder symptoms at age 9.

Variables with a significant correlational relationship with eating disorder symptoms at 12 years were entered into separate multiple regressions for boys and girls (Table 4). Variables entered into the boys' model were dietary restraint (7y), previous eating disorder symptoms (9y) and body esteem (12y). Variables entered into the girls’ model were previous eating disorder symptoms (9y), BMI (12y), depressive symptoms (12y) and body esteem (12y).

### 3.4 Prospective predictors and correlates of eating disorder symptoms at 12 years of age

To examine the combined effects of the aforementioned variables upon eating disorder symptoms at 12 years, OLS linear regression analyses were run separately for boys and girls. The resultant regression coefficients are shown in Table 4.

For boys, higher dietary restraint (7y), higher eating disorder symptoms (9y) and lower body esteem (12y) all accounted for significant variance in eating disorder symptoms at 12 years.

For girls, higher eating disorder symptoms (9y), higher depression (12 y) and lower body esteem (12y) all accounted for variance in eating disorder symptoms at 12 years of age, but BMI (12y) did not. The initial contribution of BMI (12y) to variance in eating disorder symptoms (12y; *β* = 0.15, *p* = 0.001) was fully cancelled out by the addition of body esteem (12y) to the model, rendering the contribution of BMI non-significant.

The final regression model accounted for a greater proportion of variance in eating disorder symptoms at 12 years of age in girls (*R*^2^ = 0.49) than in boys (*R*^2^ = 0.26).

### 3.5 Multicollinearity

To examine the extent to which the regression models evidenced multicollinearity, tolerance and variance inflation factor (VIF) scores were inspected for all variables in the final models. In the boys' model, tolerance values ranged from 0.93 to 0.95 and VIF values ranged from 1.05 to 1.08, suggesting that multicollinearity was not a concern. In the girls' model, tolerance values ranged from 0.49 to 0.86 and VIF values ranged from 1.17 to 2.03. The relationship between BMI (12y) and body esteem (12y), as shown in Table 3, likely accounts for these less optimal collinearity statistics, even though the latter statistics are within the acceptable range (O'Brien, 2007). Because BMI (12y) did not account for significant variance in girls' eating disorder symptoms, its removal from the model reduced the range of tolerance values without reducing the *R*^2^ value at all. The revised range for VIF was from 0.63 to 0.86 and, for tolerance, from 1.15 to 1.66.

## 4 Discussion

This study shows that higher eating disorder symptoms at 9 years significantly predicted higher eating disorder symptoms at 12 years for both boys and girls, whilst greater dietary restraint at 7 years was a significant predictor for boys. Lower 12 year body esteem (for boys and girls) and higher 12 year depressive symptoms (for girls) were also associated with higher 12 year eating disorder symptoms. A number of variables, which had been included based on adult risk factors for disordered eating, did not function as predictors of eating disorder symptoms, notably previous body dissatisfaction and concurrent BMI. Low body esteem appeared to have developed alongside eating disorder symptoms rather than acting as a predictor. Girls’ body esteem at 12 years fully accounted for the initially observed univariate relationship between 12 year BMI and eating disorder symptoms.

Initial eating disorder symptom score at 9 years of age was the strongest predictor of subsequent eating disorder symptoms, a finding consistent with the overwhelming majority of previous studies of children and young adolescents (Keel et al., 1997, Wichstrøm, 2000). Such attitudes are moderately stable over time, appearing to belie assertions that children's eating disorder symptoms are temporally and conceptually unstable (Micali & House, 2011). This finding reinforces the importance of targeting children with higher eating disorder symptoms in preadolescence for possible intervention before they enter adolescence itself.

In keeping with initial hypotheses and previous research (Allen et al., 2008, Gardner et al., 2000, Keel et al., 1997, Parkinson et al., 2012), prior body dissatisfaction did not prospectively predict higher eating disorder symptoms in this cohort, whereas concurrent body esteem at 12 years old did. This provides additional weight to the premise that body dissatisfaction is not a reliable causal risk factor for eating disorder symptoms in childhood. The significant associations between lower body esteem and higher eating disorder symptoms at 12 years of age for both boys and girls suggest that body dissatisfaction may co-develop with eating disorder symptoms in preadolescence rather than the former preceding the latter. Similar prospective findings have been obtained previously (Smolak, 2009, Wertheim et al., 2009) although only one study – involving an older sample - gathered data over a comparable 6-year period (Ferreiro et al., 2012).

A clear sex difference was found in this cohort in depressive symptoms and their association with eating disorder symptoms at age 12; concurrent depressive symptom scores were a highly significant correlate of higher eating disorder symptoms at 12 years of age in girls but not boys. This fits with previous findings that sex differences in links between depressive and disordered eating symptoms emerge around the age of 13 years (Ferreiro et al. 2012). It has been proposed that at this age, girls but not boys are more likely to use disordered eating behaviours to relieve depressive symptoms and, reciprocally, disordered eating gives rise to negative self-evaluations and depressed affect (Beato-Fernández et al., 2007). In the current study, boys’ depressive symptom scores were low and showed little variance; this may also explain the absence of a significant effect in the predictive model.

Conversely, dietary restraint at 7 years directly predicted subsequent eating disorder symptoms at 12 years for boys but not girls, despite predicting eating disorder symptoms for children of both sexes at the earlier 9 year follow up (Parkinson et al., 2012). This suggests that early dietary restraint may play a more pivotal role in developing eating disorder symptoms in boys than girls. Notably, however, the final regression model for girls accounted for almost twice as much variance in eating disorder symptoms as the model for boys, suggesting that additional, untested predictive variables may have been missing from the boys' model. Such variables may include the pursuit of muscularity and athletic internalisation, which reflect the male ideal body more closely than the ‘thin ideal’ (Rodgers, Ganchou, Franko, & Chabrol, 2012). However, the overall proportion of variance accounted for by the predictive models for boys and girls (26% vs 49% respectively) compares favourably to that of previous studies with slightly older children, using similar variables (Ferreiro et al., 2012). These findings suggest that preventative measures against both depression and disordered eating need to take into account the sex differences on internalising symptoms found in this cohort and previously.

The dual pathway model, based on research with older populations, suggests that dietary restraint and depression provide two routes via which body dissatisfaction is enacted through behavioural and cognitive symptoms of disordered eating (Stice & Agras, 1998). In the current sample, this was not found to be the case: dietary restraint acted as a predictor of eating disorder symptoms in boys but not girls, whilst depressive symptoms had an effect on girls' eating disorder symptoms but not boys'. Similar relationships have been found before in adolescent populations, in relation to specific subsets of eating disorder symptoms, but patterns of sex differences vary (Field et al., 1999, Ricciardelli and McCabe, 2001). It is plausible that these variables act as specific risk factors at different points throughout childhood and adolescence, and that their influence may, to some extent, depend upon the child's sex.

Regarding gender differences in levels of eating disorder symptoms, older adolescents' eating disorder symptoms are typically higher in girls than boys (Ferreiro et al., 2014). In this cohort at 9 years, boys' eating disorder symptoms exceeded girls' (Parkinson et al., 2012) whereas at 12 years we have found no significant difference between their scores. This finding is consistent with previous research in which gender differences emerged around 13 years of age (Ferreiro et al., 2012, Rolland et al., 1997). Over the time between the 9 and 12 year assessments, both girls' and boys’ eating disorder symptom scores decreased, a trend seen in several similar studies (e.g., Combs et al., 2012). Others have noted age-related increases in girls but not boys (Gardner et al., 2000) or found age-related declines in boys but not girls (Ferreiro et al., 2011, Ferreiro et al., 2014).

Evidence in this field increasingly suggests that preadolescent psychological variables provide a critical context within which the seismic changes of puberty occur. Yet there remains a lack of longitudinal research into the childhood antecedents of eating disorder symptoms in early adolescence, despite the physical and psychological developmental risks these phenomena pose. Our research presents a picture of these antecedents at 12 years of age, alongside within-time correlates, using data gathered at 7 years, 9 and 12 years (Parkinson et al., 2012). It benefits from a unique, representative UK population-based birth cohort of boys and girls, making the findings generalisable to similar populations. It focuses on children aged 7–12 years of age, a key developmental window for understanding eating psychopathology (Davison et al., 2003, Smolak, 2004, Smolak, 2009, Wertheim et al., 2009). Indeed, a recent review highlighted the importance of longitudinal research, involving boys, into causal risk factors for sub-syndromal disordered eating over the preadolescent period: the current study meets all of these criteria (Culbert et al., 2015). No previous study has drawn on data from three time-points spanning preadolescence, spaced over six years, addressing this particular constellation of empirically-justifiable risk factors. However, the current study has several limitations. First, sample attrition may have introduced biases over time, although lower socio-economic families were over-represented in the initial cohort, and attrition has been higher in such families, making the remaining cohort more representative over time (Parkinson et al., 2010). The current sample therefore remains socially diverse and representative of the north of England. Second, use of self-report instruments, specifically depressive symptoms and eating disorder symptoms, may have led to under or over-reporting of symptoms; this is a particular hazard with young children (Erickson & Gerstle, 2007). Third, key putative sociocultural predictors of eating disorder symptoms – such as thin-ideal internalisation and perceived pressure to be thin – were not measured (Evans et al., 2013); it is possible that such unmeasured latent variables account for some of the findings.

Future research should seek to theoretically model the relationships among intrapersonal predictors and correlates of disordered eating, along sociocultural and biological factors over the developmental course of pre- and early-adolescence. This could be operationalised through a framework such as the biopsychosocial model, and would enable a more nuanced understanding of developmental ‘cause’ in this context (Rodgers et al., 2014). A key question to address will be whether body dissatisfaction functions as a causal risk factor in childhood for subsequent eating disorder symptoms. Evidence from the current study and others (e.g., Ferreiro et al., 2012) suggests not. However, to conclusively answer this question, additional studies with similar preadolescent age groups are needed which adopt Stice’s (2002) recommendations to adjust for initial levels of the outcome variable (and, preferably, subsequent levels of the predictor variable) when attempting to ascertain temporal precedence, as done in studies of this cohort (Parkinson et al., 2012). Consideration should also be given to different theoretical models for the development of boys' eating disorder symptoms, since most existing models draw on data from adolescent female samples and focus upon pressure to be thin, rather than pressures towards hyper-muscularity as well (Culbert et al., 2015).

In summary, the findings of this study add to the small but growing body of prospective research into the emergence and consolidation of eating disorder symptoms in children and young adolescents, taking into account sex differences. Such research has the potential to inform an understanding of factors that place children at the greatest risk of disordered eating, paving the way towards effective interventions (Pratt & Woolfenden, 2002). Our findings strongly suggest the importance of early interventions (before the age of 9 years) to address children's eating disorder symptoms, since a higher level of symptoms at 9 years of age was the strongest risk factor for a higher level of symptoms at 12 years old. Efforts might be profitably aligned with interventions to prevent excess weight gain and/or depression, given the behavioural and possible aetiological overlap between these phenomena (Haines and Neumark-Sztainer, 2006, Neumark-Sztainer, 2005). In particular, our results indicate that a focus on childhood dietary restraint in boys, on body dissatisfaction concurrent with eating disorder symptoms for both boys and girls, and on concurrent depression (in girls) in early adolescence may help identify children with a phenotype suggestive of an elevated risk for future eating disorder symptoms.

## Conflicts of interest

The authors have no conflicts of interest to declare.

## Figures and Tables

**Table 1:** Representativeness of original cohort and subsequent attrition.

	Baseline (n = 1011)^b^	12 years (n = 505)^c^
	n (%)	n (%)
Townsend quintile^a^		
1 (most affluent)	156 (15)	95 (19)
2	204 (20)	110 (22)
3	227 (23)	113 (22)
4	226 (22)	96 (19)
5 (least affluent)	192 (19)	85 (17)
Missing	6 (1)	6 (1)

a Based on Townsend deprivation index from 1991 UK census, using enumeration districts as the unit of analysis with the northern region of England as the population for comparison for the calculation of the quintiles.

b 18 sets of twins.

c 11 sets of twins.

**Table 2:** Median values for main variables and sex differences (N = 516).

	Boys (n = 254)		Girls (n = 262)			
	n (%)	Median (IQR)	n (%)	Median (IQR)	Z	*p*
**7 years**:						
BMI	211 (83.1)	16.1 (2.4)	281 (83.2)	16.3 (2.7)	−0.9	0.349
BMI *z*-score	211 (83.1)	0.4 (1.5)	281 (83.2)	0.3 (1.3)	−1.5	0.148
Dietary restraint	206 (81.1)	2.0 (0.6)	214 (81.7)	2.1 (0.6)	−0.7	0.476
Body dissatisfaction	205 (80.7)	0.0 (1.0)	208 (79.4)	−1.0 (2.0)	−2.8	**0.005**
**9 years**:						
BMI	226 (89.0)	17.1 (2.8)	236 (90.1)	17.6 (3.4)	−2.0	0.042
BMI *z*-score	226 (89.0)	0.6 (1.5)	236 (90.1)	0.5 (1.4)	−1.3	0.199
Eating disorder symptoms	216 (85.5)	12.0 (12.8)	233 (88.9)	11.0 (14.5)	1.9	0.053
Body dissatisfaction	224 (88.2)	0.0 (1.0)	235 (89.7)	−1.0 (1.0)	−2.7	**0.007**
**12 years**:						
BMI	251 (98.8)	19.6 (4.5)	259 (98.9)	20.2 (4.6)	−2.2	0.028
BMI *z*-score	251 (98.8)	0.7 (1.6)	259 (98.9)	0.7 (1.6)	-0.80	0.421
Eating disorder symptoms	254 (100.0)	6.0 (6.0)	262 (100.0)	6.0 (8.0)	−0.1	0.882
Depressive symptoms	254 (100.0)	1.0 (2.0)	259 (98.9)	1.0 (4.0)	−3.3	**0.001**
Body esteem	253 (99.6)	100.0 (5.6)	262 (100.0)	94.4 (22.2)	−6.1	**<0.001**

Boldface denotes significance at *p* ≤ 0.005. IQR, interquartile range; BMI, body mass index; n, number of participants for whom data on the specified variable was available.

**Table 3:** Correlations among eating disorder symptoms at 12 years of age and other key variables.

	BMI (12 y)	Eating disorder symptoms (12y)	Depressive symptoms (12y)	Body esteem (12 y)	BMI (9 y)	Eating disorder symptoms (9y)	Body dissatisfaction (9y)	BMI (7y)	Dietary restraint (7y)	Body dissatisfaction (7y)
BMI 12 y	–	**0.23**	0.14	**-0.39**	**0.85**	0.17	**0.44**	**0.77**	0.19	**0.30**
Eating disorder symptoms (12y)	0.07	–	**0.54**	**-0.51**	0.18	**0.37**	0.11	0.18	0.03	-0.05
Depressive symptoms (12y)	0.09	0.15	–	**-0.59**	0.14	**0.33**	0.11	**0.22**	0.03	0.03
Body esteem (12y)	**-0.39**	**-0.28**	**-0.44**	**-**	**-0.34**	**-0.23**	-0.19	**-0.35**	-0.01	-0.04
BMI (9y)	**0.84**	0.06	0.09	**-0.30**	–	0.11	**0.52**	**0.89**	0.08	**0.35**
Eating disorder symptoms (9y)	0.13	**0.36**	**0.21**	**-0.21**	0.05	–	**0.29**	0.16	0.11	0.08
Body dissatisfaction (9y)	**0.39**	0.07	0.20	**0.26**	**0.46**	0.17	–	**0.46**	0.06	0.19
BMI (7y)	**0.74**	0.03	0.05	**0.22**	**0.88**	-0.03	**0.40**	–	0.08	**0.35**
Dietary restraint (7y)	0.15	**0.21**	0.09	-0.15	0.18	0.13	0.13	0.12	–	0.13
Body dissatisfaction (7y)	**0.25**	0.01	0.01	-0.09	**0.25**	-0.02	**0.26**	**0.32**	0.09	–

Correlations are Spearman's *ρ*. Coefficients for boys are shown below the diagonal, and coefficients for girls are shown above the diagonal.

Boldface signifies *p* ≤ 0.005.

**Table 4:** Multivariate regressions of eating disorder symptoms at 12 years on independent variables by sex: final models.

	Unstandardised coefficients		Standardised coefficients	*t*	*p*	*R*^*2*^
	*B*	*SE*	*β*			
**a) Boys**						
**7 years:**						
Dietary restraint	2.00	0.88	0.15	2.28	**0.020**	
**9 years:**						
Eating disorder symptoms	0.11	0.04	0.18	2.76	**0.006**	
**12 years:**						
Body esteem	−0.18	0.03	−0.39	−5.93	**<0.001**	**0.26**
**b) Girls**						
**9 years:**						
Eating disorder symptoms	0.18	0.04	0.23	4.41	**<0.001**	
**12 years:**						
BMI (kg/m^2^)	0.02	0.12	0.01	0.18	0.860	
Depressive symptoms	0.53	0.16	0.21	3.26	**0.001**	
Body esteem	−0.18	0.03	−0.44	−6.56	**<0.001**	**0.49**

Boldface denotes significance at *p* ≤ 0.05. *B*, unadjusted regression coefficient; Eating disorder symptoms, Children's Eating Attitudes Test; Depressive symptoms, Child Depression Inventory – Short form.
